# Longitudinal gut microbiota development from infancy to toddlerhood: a systematic review and platform-stratified meta-analysis

**DOI:** 10.3389/fped.2026.1898215

**Published:** 2026-08-18

**Authors:** Hongyun Li, Qinghong Xia

**Affiliations:** 1 Operating Room of Anesthesia Surgery Center, West China Hospital, Sichuan University, Chengdu, China; 2 West China School of Nursing, Sichuan University, Chengdu, China; 3 Operating Room, West China Hospital, Sichuan University, Chengdu, Sichuan, China

**Keywords:** gut microbiota, infant, longitudinal cohort, meta-analysis, microbial maturation, toddlerhood

## Abstract

**Background:**

Gut-microbiota development during early life is associated with immune, metabolic, and neurodevelopmental maturation, but longitudinal findings differ across sampling schedules and sequencing platforms.

**Methods:**

PubMed/MEDLINE, Web of Science Core Collection, Embase.com, and Scopus were searched from inception through 21 July 2026. Two authors independently screened records and full reports. Reports were linked to their underlying studies/cohorts, and the full evidence set was mapped at cohort level. Hedges-corrected standardized mean changes were synthesized with REML random-effects models and modified Hartung-Knapp intervals only within compatible developmental windows, sequencing platforms, and taxonomic levels.

**Results:**

The searches identified 10,337 records; 5,640 duplicates were removed and 4,697 unique records were screened. Of 344 reports sought, 11 were not retrieved, 333 were assessed, and 173 reports representing 95 studies/cohorts were included. Seven reports contributed to the primary quantitative panels. In primary 16S syntheses, Shannon diversity was higher at approximately 12 months (Hedges g 1.62, 95% CI 0.85–2.39; I^2^ = 83.0%; k = 4), as was Chao1 richness (g 2.14, 95% CI 1.69–2.60; I^2^ = 0%; k = 3). Exact-paired-only sensitivity estimates remained positive but were imprecise for Shannon (g 1.93, 95% CI −1.00–4.85; k = 2) and Chao1 (g 2.07, 95% CI −1.73–5.88; k = 2). Primary taxon syntheses contained two 16S estimates per outcome and were exploratory.

**Conclusions:**

The evidence suggests that alpha diversity generally increases during the first year of life, although the magnitude and between-cohort consistency remain uncertain. Certainty ranged from low to very low, and taxon-specific findings require platform- and age-window-specific interpretation.

## Introduction

The human gut microbiota plays a fundamental role in early life development, influencing immune maturation, metabolic programming, and neurodevelopmental processes (1, 2). The initial establishment and subsequent succession of the gut microbiota during infancy and early childhood represent a critical window for shaping long-term health outcomes (3, 4). Disruptions in microbial colonization during this period have been linked to obesity, allergies, asthma, and neurodevelopmental disorders (5–7). Numerous cohort studies have tracked gut microbiota development in early life using high-throughput sequencing to characterize microbial composition over time (8, 9). These studies show rapid change during the first three years, with gradual progression from a facultative-anaerobe-dominated community toward a more stable and diverse adult-like microbiota (10–12). Findings vary across sampling schedules, geography, feeding, delivery mode, antibiotic exposure, and analytic pipelines (11, 13, 14). Despite the growing literature, cross-study taxonomic comparisons remain complicated by differences in age definition, sequencing platform, and reporting format.

In this review, we performed a systematic review and meta-analysis of published longitudinal cohort studies investigating gut microbiota development during the first two years of life. We aimed to quantify within-study changes in alpha diversity and selected dominant taxa across explicitly reported sampling ages, while accounting for differences in sequencing platform and developmental window.

## Methods

### Literature search strategy

PubMed/MEDLINE, Web of Science Core Collection, Embase.com, and Scopus were searched from database inception through 21 July 2026. Database-specific strategies combined controlled vocabulary and free-text terms for newborns, infants, toddlers, gut microbiota or microbiome, and longitudinal, cohort, follow-up, or repeated-measures designs (Supplementary Table S1). No date, language, document-type, or subject-area filters were applied. The searches returned 2,846 PubMed, 2,167 Web of Science, 2,914 Embase, and 2,410 Scopus records. Hongyun Li and Qinghong Xia independently screened every title/abstract and every retrieved full report; disagreements were resolved through discussion. The review followed PRISMA 2020 (15). It was not prospectively registered, and no formal librarian/PRESS review was performed.

### Eligibility criteria

Studies or cohorts were eligible when they included at least 20 participants; healthy full-term infants or a separable healthy full-term subgroup; at least two within-child gut-microbiota sampling times, with one in infancy and another at or after approximately 12 months but before 3 years; 16S rRNA gene or whole-metagenome shotgun sequencing; and bacterial-community, alpha-diversity, or taxonomic-abundance outcomes. Eligible designs comprised longitudinal natural-history cohorts and separable observational components of broader studies. Preterm-only or disease-only populations without an eligible subgroup, non-separable interventions, cross-sectional or single-time-point designs, non-sequencing studies, samples below the participant threshold, conference abstracts, reviews, and editorials were excluded. Reports not retrieved after documented attempts were recorded separately and were not assigned an eligibility decision. Multiple publications from one cohort were retained as companion reports but linked to one cohort identifier. Reports describing more than one eligible cohort were linked to every contributing cohort, while each underlying cohort was counted once.

### Study selection and data extraction

For each retained report, we extracted bibliographic information, country or setting, eligible participant count, repeated or paired denominator when reported, sampling ages, sequencing platform, diversity or taxonomic outcome, and the availability of quantitative data. Hongyun Li performed the structured extraction and report to cohort linkage, and Qinghong Xia independently verified each completed record; discrepancies were resolved through discussion. Multiple reports from the same underlying cohort were linked to one study identifier and were not treated as independent participant samples.

Risk of bias and certainty of evidence. Hongyun Li and Qinghong Xia independently applied the JBI cohort checklist at the study/cohort level (16) and resolved disagreements through discussion. Unclear was retained when reporting was insufficient, and items not applicable to within-child developmental outcomes were marked N/A. Domain-level consensus judgments for all 95 cohorts are provided in Supplementary Table S8. Certainty was evaluated by outcome using GRADE guidance for longitudinal natural-history and prognostic-factor evidence (17). A conventional observational starting point was retained as a framework sensitivity analysis (Supplementary Table S9). Because every synthesis contained fewer than 10 estimates, funnel-plot tests were not performed. Small-study and analyzable-result availability were considered qualitatively within the publication-bias and imprecision domains.

### Data synthesis

Continuous longitudinal outcomes were expressed as Hedges-corrected standardized mean change for repeated measures (SMCRP) (18), oriented so that positive values indicate higher values at the later age. All participant-level inputs used for effect-size reconstruction had previously been published and released as article-associated source or supplementary data; no unpublished, investigator-supplied, or newly collected participant data were used. Random-effects models used REML with modified Hartung-Knapp intervals (19) and were restricted to compatible platforms, taxonomic levels, and developmental windows. Participant-level paired estimates were calculated for Carlson et al. (20), Dutton et al. (21), Bäckhed et al. (9), and Selma-Royo et al. (22). Azad et al. (23) and Moroishi et al. (24) contributed published visit-level moments; r = 0.50 was used for display and r = 0.00, 0.25, 0.50, and 0.75 were examined. Exact-paired-only sensitivity analyses excluded published marginal-moment estimates. For SKOT I (25), dispersion was estimated from published box-and-whisker summaries. Values digitized from the Sumedang 6–12 month boxplots (26) were used only in a secondary expanded-window sensitivity analysis. Family-level Bifidobacteriaceae was not pooled with genus-level Bifidobacterium. Taxon models summarized standardized changes in study-reported relative abundance. Standardization harmonizes scale but does not remove compositional closure; without consistently reported absolute loads or log-ratio data, taxon effects were interpreted as relative compositional shifts rather than changes in absolute organism abundance. Effect inputs and derivations are reported in Supplementary Table S6, with all meta-analysis and sensitivity results in Supplementary Table S7. Robustness analyses included exact-paired-only, leave-one-out, within-person correlation-grid, HEAL no-malaria-restriction, platform-stratified, and secondary expanded-window analyses. Analyses were conducted in Python 3.12 using prespecified custom implementations of the reported estimators.

## Results

### Overview of included studies

**Figure 1:**
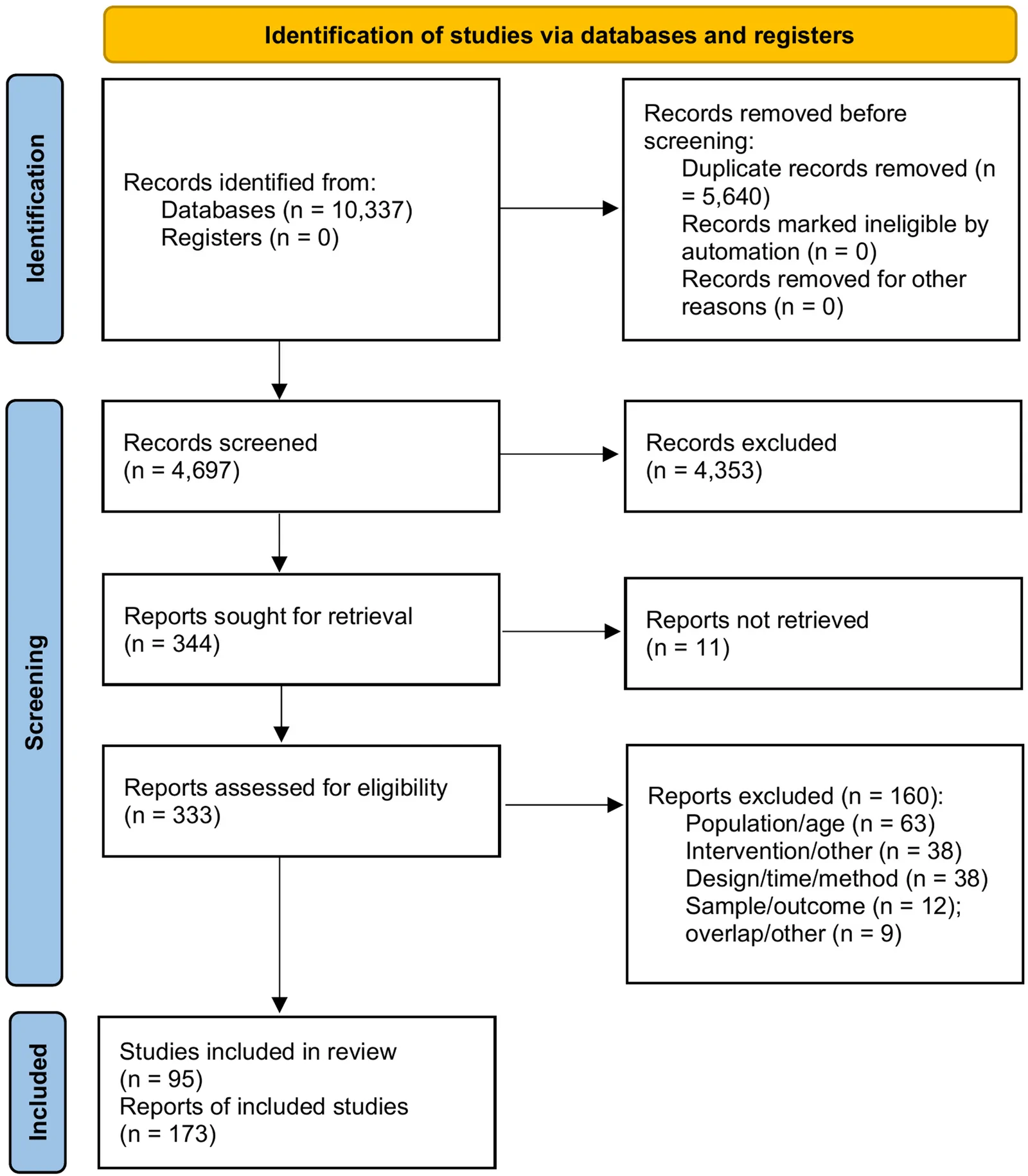
PRISMA 2020 flow diagram for the four-database search and study-selection process. Reports not retrieved after documented attempts are reported separately and were not reclassified as full-text exclusions.

The four database searches identified 10,337 records: PubMed (*n* = 2,846), Web of Science (*n* = 2,167), Embase (*n* = 2,914), and Scopus (*n* = 2,410). After removal of 5,640 duplicates, 4,697 unique records underwent title and abstract screening and 4,353 were excluded. Of 344 reports sought for retrieval, 11 were not retrieved; 333 full reports were assessed, 160 were excluded with one primary reason, and 173 reports representing 95 studies/cohorts were included (Figure 1). Full-text exclusions and reports not retrieved are listed in Supplementary Table S4. Complete citations for all included reports and their cohort links are provided in Supplementary Table S3.

The 173 included reports mapped to 95 independent studies/cohorts rather than 173 independent participant samples. Directly extractable structured fields included eligible participant counts for 74 cohorts, sampling ages for 91, sequencing methods for 80, and microbiota outcomes for 90. Supplementary Table S2 retains NR for values that were not directly extractable and provides source-adjudicated longitudinal eligibility evidence for every cohort. Supplementary Table S3 identifies four reports that contributed information to more than one underlying cohort and preserves the study-counting convention.

Seven reports from seven independent cohorts contributed to the primary quantitative panels (Table 1): Carlson et al. from UNC (United States) (20), Azad et al. from CHILD (Canada) (23), Moroishi et al. from NHBCS (United States) (24), Dutton et al. from HEAL Africa (Democratic Republic of the Congo) (21), Bäckhed et al. (Sweden) (9), Selma-Royo et al. from MAMI (Spain) (22), and Laursen et al. from SKOT I (Denmark) (25). Five used 16S rRNA gene sequencing and two used shotgun metagenomics. Their displayed longitudinal denominators were 19, 167, 44, 42, 93, 25, and 114, respectively. Lawley et al. from the Indonesian Sumedang cohort contributed an eighth report only to the secondary 6–12 month expanded-window sensitivity analysis (26).

**Table 1:** Characteristics of the seven reports contributing to the primary quantitative panels.

Study/cohort	Setting	Paired n	Age contrast	Platform	Outcomes and direction at later age
Carlson et al. (20)/UNC	United States	19	1–12 months	16S rRNA (V1-V2)	Shannon and Chao1 higher; Bifidobacterium and Enterobacteriaceae lower; Lachnospiraceae higher.
Azad et al. (23)/CHILD	Canada	167	3–12 months	16S rRNA	Shannon and Chao1 higher.
Moroishi et al. (24)/NHBCS	United States	44	6 weeks to 12 months	16S rRNA	Shannon higher.
Dutton et al. (21)/HEAL Africa	DR Congo	42	6 weeks to 12 months	Full-length 16S rRNA	Shannon, Chao1, and Lachnospiraceae higher; Bifidobacterium and Enterobacteriaceae lower.
Bäckhed et al. (9)	Sweden	93	4–12 months	Shotgun metagenomics	Shannon and Lachnospiraceae higher; Bifidobacterium and Enterobacteriaceae lower.
Selma-Royo et al. (22)/MAMI	Spain	25	1–12 months	Shotgun metagenomics	Shannon and Lachnospiraceae higher; Bifidobacterium and Enterobacteriaceae lower.
Laursen et al. (25)/SKOT I	Denmark	114	9–18 months	16S rRNA (V3)	Bifidobacteriaceae and Enterobacteriaceae lower; Lachnospiraceae higher.

The JBI assessment classified 3 studies/cohorts as low concern and 92 as having some concerns; none was classified as high concern. Incomplete reporting of follow-up completeness, missing-data handling, and longitudinal statistical methods was most common. Under the longitudinal natural-history GRADE framework, certainty was low for Chao1, Enterobacteriaceae, and Lachnospiraceae and very low for Shannon and Bifidobacterium. The conventional observational-starting-point sensitivity framework yielded very-low-certainty ratings for all outcomes (Supplementary Tables S8, S9).

### Alpha diversity patterns across early life

**Figure 2:**
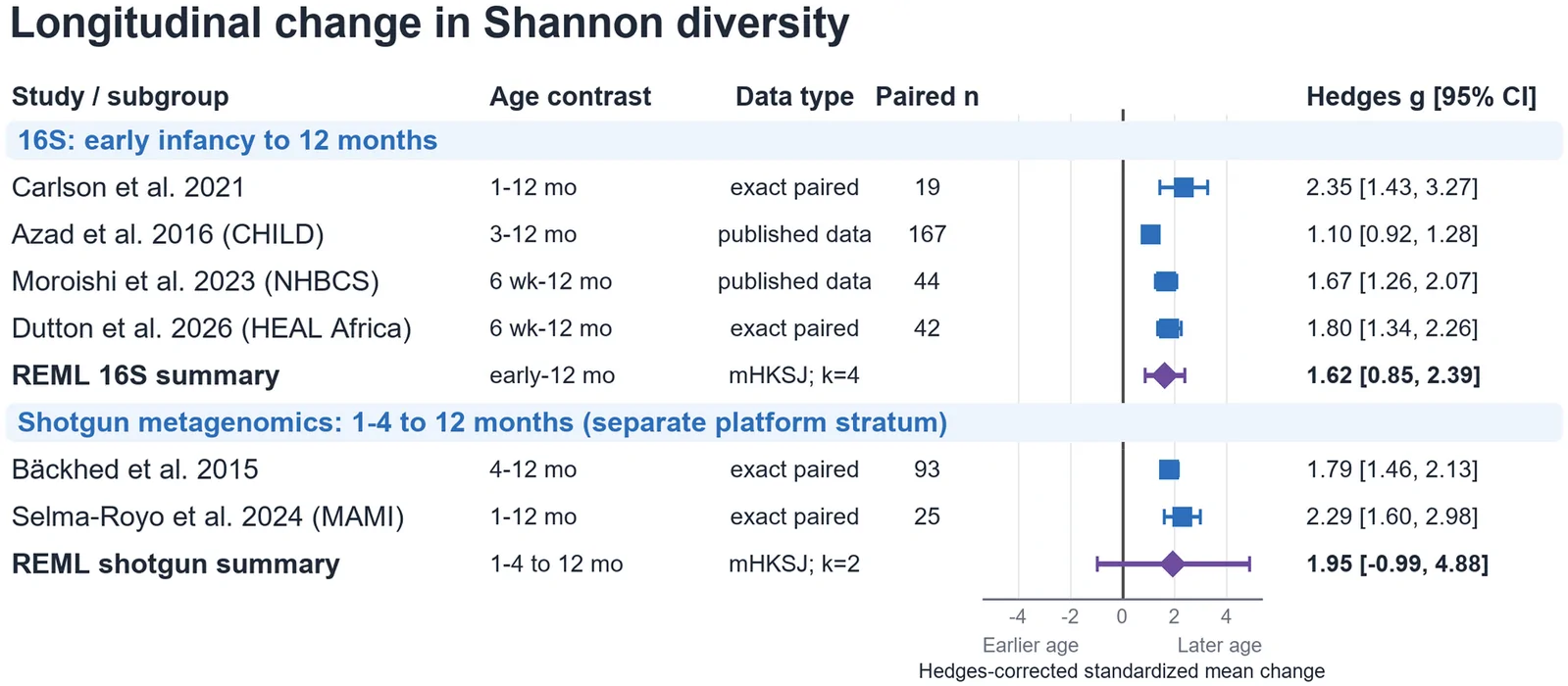
Platform-stratified longitudinal changes in Shannon diversity. The primary 16S early-infancy-to-12-month summary uses REML with a modified Hartung-Knapp interval. Carlson and HEAL Africa use exact paired data; marginal-moment rows display r = 0.50, with r = 0.00-0.75 retained in sensitivity analyses. Bäckhed (4–12 months) and MAMI (1–12 months) form a separate exact paired shotgun subgroup; no estimate combines 16S and shotgun data.

**Figure 3:**
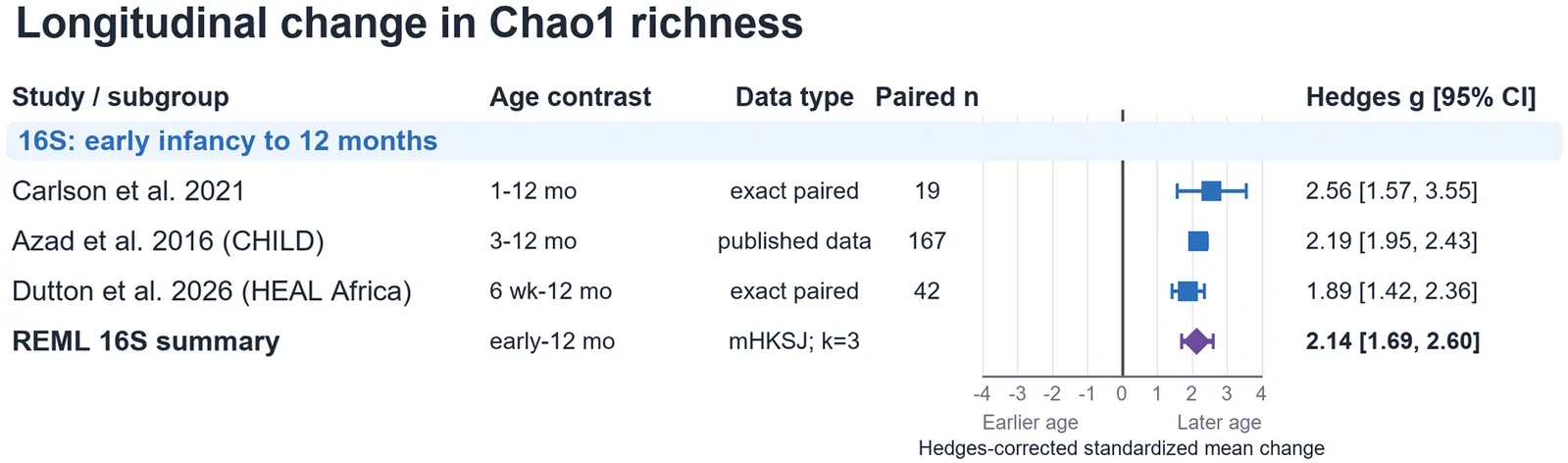
Random-effects forest plot of Chao1 richness from early infancy to 12 months. Carlson and HEAL Africa use exact paired data; the Azad/CHILD marginal-moment row displays r = 0.50, with sensitivity analyses across the specified correlation grid. REML estimates use modified Hartung-Knapp intervals.

Four independent 16S reports contributed Shannon estimates from early infancy to 12 months. The REML estimate indicated higher Shannon diversity at the later age (Hedges g 1.62, 95% CI 0.85–2.39; *τ*^2^ = 0.170; I^2^ = 83.0%; Q = 17.66, df = 3, *p* < 0.001; Figure 2). Its 95% prediction interval (−0.44–3.68) crossed the null. Bäckhed and MAMI formed a separate exact-paired shotgun subgroup (k = 2; g 1.95, 95% CI −0.99–4.88; I^2^ = 38.5%). Three 16S reports contributed Chao1 estimates (g 2.14, 95% CI 1.69–2.60; I^2^ = 0%; Figure 3). In the strict exact-paired-only sensitivity analysis, point estimates remained positive but confidence intervals crossed the null for Shannon (g 1.93, 95% CI −1.00–4.85; k = 2) and Chao1 (g 2.07, 95% CI −1.73–5.88; k = 2). Correlation-grid analyses changed the primary estimates minimally. Omitting Azad reduced Shannon I^2^ to 0%, identifying it as the principal statistical influence.

### Relative abundance of dominant taxa

The taxon panels present exploratory platform- and age-window-specific syntheses from five independent cohorts. In the primary 16S early-infancy-to-12-month subgroup, relative abundance was lower for Bifidobacterium (g −0.74, 95% CI −3.03–1.55; k = 2) and Enterobacteriaceae (g −0.74, 95% CI −3.21–1.72; k = 2) and higher for Lachnospiraceae (g 1.19, 95% CI −1.75–4.13; k = 2). Separate Bäckhed plus MAMI shotgun summaries had the same point-estimate directions but wide intervals. SKOT I 9-to-18-month 16S estimates remained separately displayed. In the secondary expanded-window 16S sensitivity analysis, addition of the Sumedang 6-to-12-month estimate gave lower Enterobacteriaceae relative abundance (g −0.61, 95% CI −1.01 to −0.22; I^2^ = 0%; k = 3) and higher Lachnospiraceae relative abundance (g 1.09, 95% CI 0.65–1.53; I^2^ = 5.3%; k = 3) at r = 0.50. The small-k prediction intervals crossed the null, and the primary age-window-specific models were unchanged.

## Discussion

This systematic review mapped 173 reports to 95 longitudinal studies/cohorts and quantitatively synthesized the subset with compatible effect data. Seven reports contributed to the primary panels, while the Sumedang report contributed only to a secondary expanded-window sensitivity analysis. Separating the full cohort evidence map from the platform-stratified meta-analysis retained the breadth of the literature without combining incompatible age windows or measurement platforms in one summary estimate.

The primary 16S analyses suggest increasing alpha diversity across the first year of life. Shannon showed substantial heterogeneity and a prediction interval that crossed the null. Chao1 was more consistent in the primary model. However, the exact-paired-only analyses for both outcomes had wide confidence intervals that crossed the null because only two cohorts remained. Thus, the positive direction is biologically plausible and consistent with microbiota maturation, but its magnitude and between-cohort reproducibility remain uncertain.

This directional pattern is consistent with age-related maturation reported in the TEDDY, Bäckhed, and Bokulich cohorts (4, 9, 13). These studies also showed that diet, antibiotics, and delivery-related exposures can alter the pace and composition of maturation. Our pooled estimates extend those observations across cohorts, but the wide Shannon prediction interval indicates that a shared direction does not imply a uniform effect size.

Developmental timing was examined using exact age contrasts rather than broad age bands. Shannon and Chao1 were pooled within the 16S early-infancy-to-12-month window, whereas early shotgun estimates and SKOT I 9-to-18-month estimates were retained as separate strata. The broader pre-12-to-12-month taxon model was evaluated as a secondary analysis with the Sumedang estimate.

**Figure 4:**
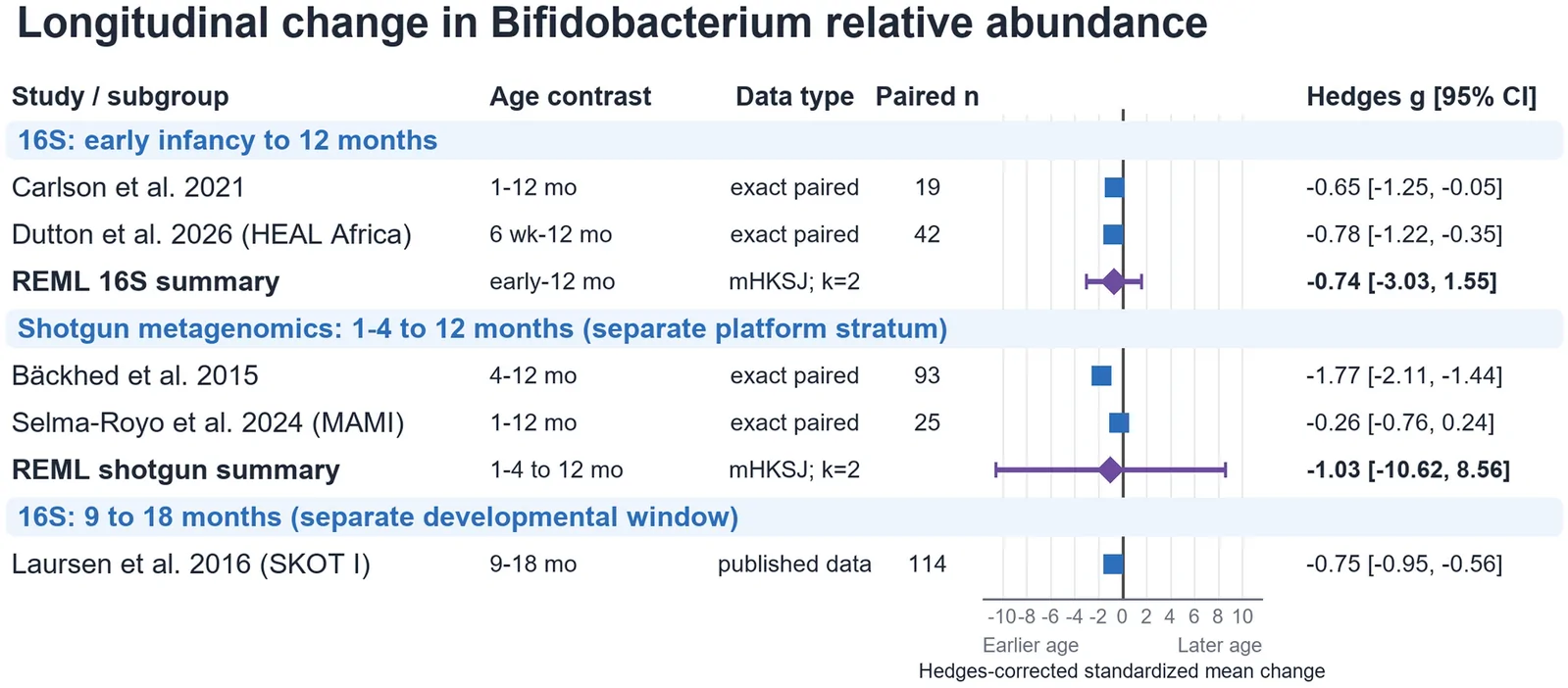
Exploratory platform- and age-window-specific change in Bifidobacterium relative abundance. Carlson and HEAL Africa form the primary 16S subgroup. Bäckhed and MAMI form a separate shotgun subgroup. The family-level SKOT I Bifidobacteriaceae estimate is shown only in the separate 9–18-month stratum. Effects describe relative compositional shifts, not absolute microbial loads.

**Figure 5:**
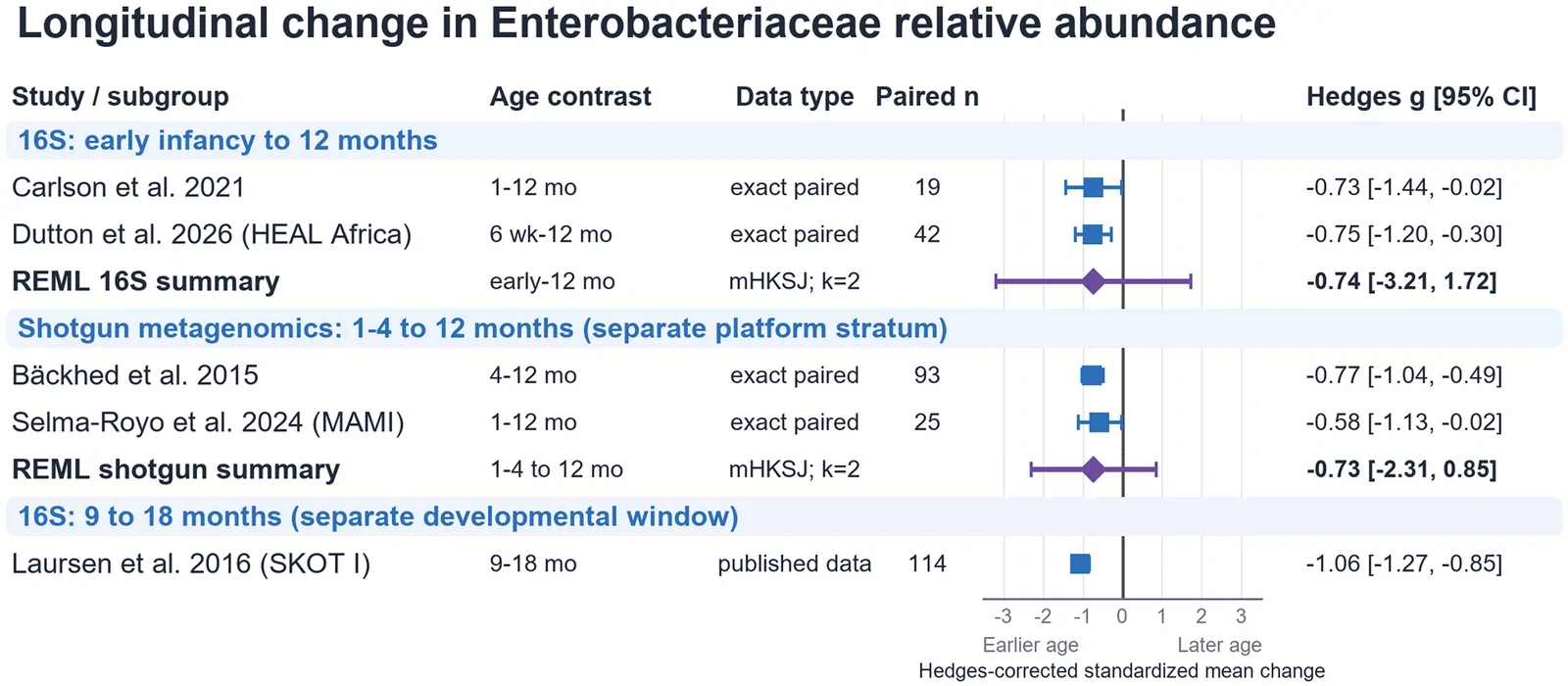
Exploratory platform- and age-window-specific change in Enterobacteriaceae relative abundance. Carlson and HEAL Africa form the primary 16S subgroup; Bäckhed and MAMI form a separate shotgun subgroup; SKOT I remains a separate 9–18-month 16S estimate. The broader-window sensitivity adds Sumedang only as a secondary analysis. Effects describe relative compositional shifts, not absolute microbial loads.

**Figure 6:**
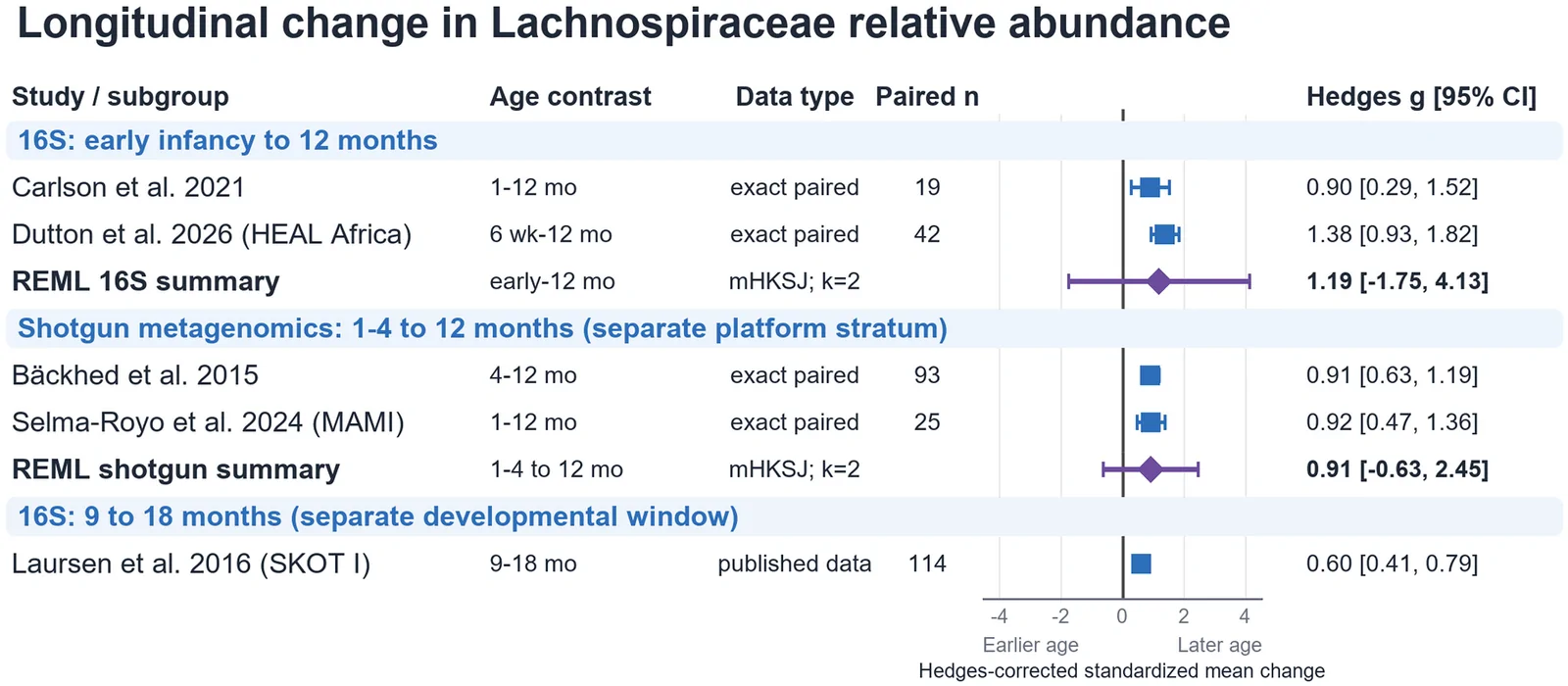
Exploratory platform- and age-window-specific change in Lachnospiraceae relative abundance. Carlson and HEAL Africa form the primary 16S subgroup; Bäckhed and MAMI form a separate shotgun subgroup; SKOT I remains a separate 9–18-month 16S estimate. The broader-window sensitivity adds Sumedang only as a secondary analysis. Effects describe relative compositional shifts, not absolute microbial loads.

Taxon-specific findings were less stable than alpha-diversity findings. The primary 16S point estimates suggested lower relative abundance of Bifidobacterium (Figure 4) and Enterobacteriaceae (Figure 5) and higher relative abundance of Lachnospiraceae (Figure 6) at the later age, but each model contained two estimates and its confidence interval included the null. The secondary expanded-window analysis strengthened the pooled Enterobacteriaceae and Lachnospiraceae signals, although its prediction intervals still crossed the null. These estimates describe relative composition. They cannot distinguish a decline in absolute taxon load from an increase in other community members.

Only seven reports provided the combination of a compatible age contrast, longitudinal denominator, central moments, and sampling variance required for the primary models. The remaining cohorts broadened the evidence map but could not be assigned quantitative directions without additional assumptions. This availability of analyzable results may favor more completely reported cohorts. It also limited formal moderator analyses for delivery mode, feeding, antibiotic exposure, geography, and analytic pipeline.

Delivery mode, breastfeeding and complementary feeding, antibiotic exposure, geography, and analytic pipeline remain important potential modifiers of early microbiota development. These variables were not pooled because their definitions and reporting were inconsistent across reports. They are considered contextual explanations for between-study variation rather than treated as causal moderators in the present analysis.

Our decision not to pool modifier effects also aligns with prior evidence. A systematic review linked prenatal and intrapartum antibiotic exposure to lower infant diversity and reduced bifidobacterial representation, while emphasizing confounding and design heterogeneity (27). Intensive sampling around the introduction of solid foods likewise associated dietary diversification with increasing Shannon diversity and richness (28). Together, these findings support treating antibiotics and complementary feeding as plausible modifiers rather than universal determinants of one developmental trajectory.

Several limitations should be considered. Eleven potentially relevant reports were not retrieved after documented attempts. Structured cohort characteristics were incompletely reported, although source-level eligibility evidence and report-to-cohort mappings are provided for all included cohorts. Geographic coverage and sample sizes were uneven, and the earliest age contrasts came from few cohorts. Shannon heterogeneity was substantial, exact-paired sensitivity analyses were imprecise, and the primary taxon models contained two estimates. Some effects relied on published marginal moments or digitized boxplots. Relative-abundance models remain subject to compositional closure, and small-study or reporting bias could not be tested formally. The review was not prospectively registered and did not undergo a formal librarian/PRESS review.

Future research should prioritize harmonized sampling protocols, standardized bioinformatic pipelines, and consistent reporting standards to enable robust cross-study comparisons. Furthermore, integration of multi-omics approaches—including metagenomics, metabolomics, and host immune profiling—may help unravel mechanistic links between microbiota composition and health outcomes. Longer-term follow-up into later childhood and adolescence will also be essential to understand the persistence or resolution of early microbial patterns over time.

## Conclusion

This systematic review and platform-stratified meta-analysis suggests that alpha diversity generally increases during the first year of life. The primary 16S models showed higher Shannon diversity and Chao1 richness near 12 months, while strict paired-data analyses retained positive point estimates with lower precision. Evidence for individual taxon trajectories was exploratory and described relative compositional change rather than absolute abundance. These findings are consistent with age-related microbiota maturation, but the magnitude and consistency of specific trajectories remain uncertain.

Future longitudinal studies using harmonized sampling schedules, complete paired reporting, and comparable bioinformatic pipelines will help refine platform- and age-specific estimates.
